# *Trypanosoma cruzi* modulates its histone post-translational modifications under nutritional stress

**DOI:** 10.3389/fcimb.2026.1941217

**Published:** 2026-09-16

**Authors:** Rafael Fogaça de Almeida, Lyris Martins Franco de Godoy

**Affiliations:** Laboratório de Biologia Molecular e Sistêmica de Tripanossomatídeos, Instituto Carlos Chagas, Fundação Oswaldo Cruz, Curitiba, Parana, Brazil

**Keywords:** histone post-translational modifications (hPTMs), histones, hPTM modulation, nutritional stress, *Trypanosoma cruzi*

## Abstract

**Introduction:**

Histone post-translational modifications (hPTMs) constitute one of the main regulatory mechanisms of chromatin state in eukaryotes. The presence of different hPTMs has been described in *Trypanosoma cruzi*; however, their roles in parasite biology and adaptation to environmental changes remain poorly understood. The nutritional stress faced by the parasite in the intestine of the invertebrate host triggers differentiation of non-infective epimastigotes into infective metacyclic trypomastigotes.

**Methods:**

The modulation of hPTMs in epimastigotes subjected to short- and long-term nutritional stress was analyzed using a bottom-up proteomic workflow based on propionic anhydride derivatization and a combination of complementary mass spectrometry-based proteomic approaches (LC-MS/MS and GeLC-MS/MS) with label-free quantification.

**Results:**

A total of 172 hPTMs were identified. Monomethylation and acetylation were the most abundant hPTM classes in all conditions, whereas dimethylation, trimethylation, and crotonylation showed significant changes under more prolonged nutritional stress. Among the identified modifications, 20 hPTM marks distributed across 16 histone peptides were differentially modulated across conditions, with most changes observed during prolonged nutritional stress. Notably, N-terminal acetylation of H2A.Z (K42–K58) increased significantly as nutrient availability decreased over time.

**Discussion:**

These results show that *T. cruzi* dynamically modulates its hPTM landscape in response to nutrient limitation.

## 1 Introduction

*Trypanosoma cruzi* is a flagellated protozoan belonging to the order Kinetoplastida and the family Trypanosomatidae and is the etiological agent of Chagas disease (Chagas, 1909). The parasite exhibits a heteroxenic life cycle involving both a triatomine insect vector and mammalian hosts, including humans, and passes through distinct developmental stages adapted to these different environments (Chagas, 1909). To adapt to these changing conditions, the parasite undergoes marked morphological and physiological changes that require rapid and efficient regulation of gene expression to generate molecules suited to each stage of its life cycle. One of the fundamental processes by which *T. cruzi* acquires infective capacity is its differentiation from the epimastigote form to the metacyclic trypomastigote form. This process occurs in the digestive tract of the triatomine vector and is known as metacyclogenesis (Camargo, 1964). Metacyclic trypomastigotes are generated under nutrient-limited conditions, a hallmark of the environment faced by the parasite in the digestive tract of the triatomine vector. *In vitro*, stationary-phase epimastigotes are considered a pre-adaptive stage for metacyclogenesis, and nutritional stress is one of the major triggers of this differentiation process (Hernández et al., 2012; Figueiredo et al., 2000). Experimentally, nutritional stress can be induced by transferring stationary-phase epimastigotes to triatomine artificial urine (TAU), a nutrient-poor medium that mimics aspects of the nutrient-limited environment encountered by epimastigotes in the triatomine intestinal tract (Contreras et al., 1985). Although metacyclogenesis has been studied from different perspectives, such as differential gene expression (Krieger et al., 1999; Yamada-Ogatta et al., 2004), proteomics (Parodi-Talice et al., 2007; de Godoy et al., 2012), and phosphoproteomics (Marchini et al., 2011; Amorim et al., 2017; Lucena et al., 2019), little is known about the key epigenetic mechanisms associated with nutritional stress, a physiological stimulus that triggers metacyclogenesis.

In *T. cruzi* and other trypanosomatids such as *T. brucei* and *Leishmania* sp., the control of gene expression occurs mainly at the post-transcriptional level (Clayton, 2019). Nevertheless, genome organization constitutes an important layer of gene expression control, particularly during stress responses and cell cycle progression (Kelly et al., 2012). Consistent with this, trypanosomatids harbor a conserved repertoire of epigenetic regulators, including histone-modifying enzymes, chromatin-remodeling complexes, histone chaperones, histone variants, specific DNA modifications, and histone post-translational modifications (hPTMs) (Martínez-Calvillo et al., 2018). In recent years, numerous hPTM marks have been characterized in the different histones of *T. cruzi* (Picchi et al., 2017; de Almeida et al., 2021; Menezes et al., 2025), *T. brucei* (Zhang et al., 2020; Maree et al., 2022), *T. evansi* (Zhang et al., 2020), and *Leishmania braziliensis* (de Almeida et al., 2025), as well as some of their writers and erasers (Jacquet et al., 2025). These studies have shown that hPTMs are widely distributed in the canonical and variant histones of these parasites, reinforcing the hypothesis of functional conservation of these epigenetic mechanisms. However, the functional significance and contribution of most hPTMs to biological processes such as gene expression regulation, chromatin organization, DNA repair and replication, cell cycle control, and cell differentiation are still poorly or not elucidated in these organisms.

Considering that nutritional stress is one of the main physiological stimuli associated with the early adaptive events preceding metacyclogenesis, the identification of hPTMs responsive to nutrient limitation provides insights into candidate chromatin-associated responses involved in parasite adaptation under conditions linked with this life-cycle transition. In this context, the present work investigated the dynamic modulation of different hPTMs during nutritional stress in *T. cruzi*, aiming to identify changes in these epigenetic marks associated with this physiological stimulus. Using complementary mass spectrometry-based proteomics approaches, our results reveal that nutritional stress induces selective rather than global remodeling of the hPTM landscape, with prominent changes affecting histone H2A.Z acetylation and specific acetylated, methylated, and crotonylated histone peptides. These findings provide new insights into hPTM modulation in epimastigotes under nutritional stress and establish a valuable resource for future studies investigating the molecular mechanisms underlying parasite adaptation to nutrient limitation and differentiation in *T. cruzi*.

## 2 Materials and methods

The experimental strategy used to analyze *T. cruzi* hPTM modulation during nutritional stress was based on previously published studies (Picchi et al., 2017; de Almeida et al., 2021) with some modifications (**Figure 1**). Briefly, histone-enriched extracts were obtained from cells under four different biological conditions in biological triplicate, then processed using two complementary preparation strategies: a gel-based approach (GeLC-MS/MS) and a gel-free approach (LC-MS/MS). The use of both workflows was based on previous hPTM studies in which gel-based and gel-free MS sample-preparation strategies provided complementary hPTM identifications (Picchi et al., 2017; de Almeida et al., 2021). Because these workflows differ in sample processing and separation, they can provide distinct peptide coverage and improve the detection of modified histone peptides. In both preparations, histones were derivatized to block lysine sites before digestion with trypsin. The tryptic peptides were analyzed by nanoLC-MS/MS, and the resulting data were processed using an *in silico* workflow optimized for hPTM analysis.

**Figure 1:**
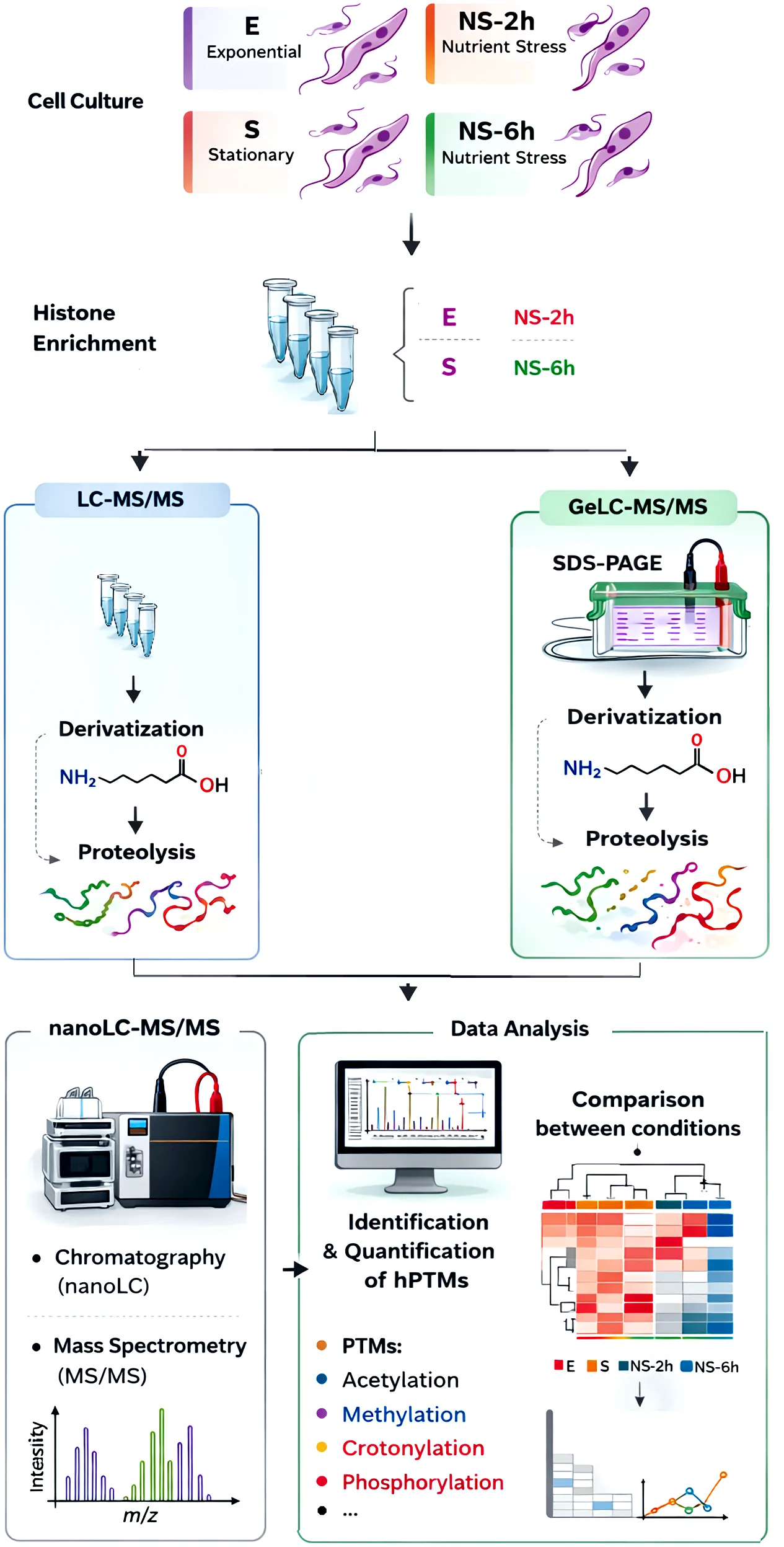
Experimental workflow for the analysis of *T. cruzi* histone post-translational modification (hPTM) modulation under different growth and nutritional stress conditions. Epimastigote cells of *T. cruzi* Dm28c were analyzed under four biological conditions: exponential growth phase (E), stationary growth phase (S), and nutritional stress for 2 h (NS-2h) and nutritional stress for 6 h (NS-6h). Histones were enriched by acid extraction from three replicates (1 × 10^9^ cells each). The histone-enriched extracts were either directly derivatized and digested in-solution with trypsin (LC-MS/MS) or first resolved by SDS-PAGE, followed by in-gel derivatization and tryptic digestion, and peptide extraction (GeLC-MS/MS). Peptides obtained from both workflows were analyzed by nanoliquid chromatography coupled with mass spectrometry (nanoLC-MS/MS). hPTMs were identified using PEAKS Studio X (v. 10), and the resulting data were subjected to quantitative and downstream comparative analyses using various analytical tools. LC-MS/MS and GeLC-MS/MS were used as complementary workflows to improve hPTM coverage and reinforce identifications shared between both strategies.

### 2.1 Cell culture

Epimastigotes of *T. cruzi* Dm28c in the exponential growth phase were cultured in liver infusion tryptose (LIT) medium at 28 °C without agitation (Camargo, 1964). Cultures were maintained by successive passages initiated at 1 × 10^6^ cells/mL. Epimastigotes were analyzed under four experimental conditions: exponential growth phase (3 days of culture; E), stationary growth phase (5 days of culture; S), and nutritional stress for 2 h (NS-2h) and 6 h (NS-6h). For the E and S conditions, parasites were collected directly from cultures in the exponential and stationary growth phases, respectively. For the nutritional stress conditions, stationary-phase parasites were washed in triatomine artificial urine (TAU) medium (190 mM NaCl, 17 mM KCl, 2 mM MgCl_2_, 2 mM CaCl_2_, and 8 mM phosphate buffer, pH 6.0) (Contreras et al., 1985), and incubated in the same medium for 2 h (NS-2h) or 6 h (NS-6h) at 28 °C. This procedure was used to induce general nutrient limitation rather than selective deprivation of a specific nutrient class. For each condition, three replicates containing 1 × 10^9^ cells each were collected by centrifugation at 5000 × g for 10 min at 4 °C.

### 2.2 Histone extraction and enrichment

The extraction and enrichment of histones were performed as previously described (Picchi et al., 2017; de Almeida et al., 2021), with modifications. Briefly, cells from each replicate were resuspended in extraction buffer A (250 mM Sucrose; 1 mM EDTA; 3 mM CaCl2; 10 mM Tris-HCl pH 7.4; 0.5% (v/v) Saponin; protease inhibitor (Complete mini EDTA-free, Roche)) and vortexed. Following centrifugation at 6000 × g for 10 min at 4 °C, the pellet was washed with extraction buffer B (extraction buffer A without saponin), centrifuged again under the same conditions, and the resulting pellet was resuspended in extraction buffer C (1% (v/v) Triton X-100; 150 mM NaCl; 25 mM EDTA; 10 mM Tris-HCl pH 8.0; protease inhibitor (Complete mini EDTA free, Roche)). The samples were centrifuged at 12000 × g for 20 min at 4 °C, and the pellet was washed three times with 100 mM Tris-HCl, pH 8.0, with centrifugation at 6000 × g for 10 min at 4 °C between washes. The final pellet was resuspended in 1 mL of 0.4 N HCl and incubated overnight at 4 °C with agitation. After centrifugation at 10000 × g for 15 min at 4 °C, the supernatant was collected, mixed with eight volumes of acetone, and incubated overnight at -20 °C. Precipitated proteins were pelleted by centrifugation at 3100 × g for 15 min at 4 °C. After careful removal of the acetone, the pellet was washed three times with 1 mL of acetone and resuspended in water. Protein concentration was then determined using the Bradford assay (Bradford, 1976). Subsequently, the enriched histone extracts were either resolved by 15% SDS-PAGE and stained with Coomassie Blue R250 or processed directly in solution, as described below.

### 2.3 Histone derivatization and proteolysis in gel (GeLC-MS/MS)

Histone bands from each replicate were excised from the gel and processed as previously described (Völker-Albert et al., 2018) with modifications. Briefly, gel pieces were destained by washing twice with 25 mM NH_4_HCO_3_ in 50% ethanol, shaking in a thermomixer at 800 rpm, for 10 min at 25 °C, followed by overnight incubation under the same conditions. After removal of the supernatant, the gel pieces were dehydrated with 100% ethanol under the same conditions. For derivatization, the gel fragments were rehydrated in 100 mM NH_4_HCO_3_, followed by the addition of propionylation solution (propionic anhydride in 100 mM NH_4_HCO_3_, 1:10) (Völker-Albert et al., 2018). The pH of the samples was maintained at 8.0. Next, 1 M NH_4_HCO_3_ was added, and the samples were incubated at room temperature for 1 h. Propionylation was repeated with fresh propionylation solution. The gel fragments were washed with 100 mM NH_4_HCO_3_, followed by ultrapure water (ddH_2_O) and 50% acetonitrile (ACN), with agitation in a thermomixer at 1300 rpm and 37 °C. The gel fragments were dehydrated with 100% ACN, the supernatant was discarded, and the samples were dried in a SpeedVac. The propionylated gel samples were then processed for nanoLC-MS/MS analysis according to a previously described protocol (Shevchenko et al., 2006). Gel pieces were incubated at 800 rpm and 25 °C for 20 min, first with digestion buffer (50 mM NH_4_HCO_3_) and then with 100% ethanol. The digestion buffer step was repeated once, and the ethanol step was repeated twice, with the supernatant discarded after each incubation. The samples were concentrated in a SpeedVac until completely dry. Trypsin solution (12.5 ng/µL trypsin in 50 mM NH_4_HCO_3_) and digestion buffer were added to the gel pieces, which were then incubated at 37 °C for 16 h. The resulting supernatant was transferred to a new tube, and peptides were extracted from the gel pieces using extraction solution (trifluoroacetic acid 3% and ACN 30%) with agitation at 800 rpm at 25 °C for 10 min. The extraction was repeated once, and the supernatants were pooled. The gel pieces were then covered with ACN (100%), incubated under the same conditions for 10 min, and the supernatant was collected. This extraction was also repeated once, and the resulting supernatants were combined with the previous peptide extracts. The sample was concentrated to approximately 10% of its original volume in a SpeedVac. The peptides were desalted onto StageTips C18 (Rappsilber et al, 2007) for analysis by nanoLC-MS/MS.

### 2.4 Histone derivatization and proteolysis in solution (LC-MS/MS)

Histone-enriched extracts were derivatized in solution with propionic anhydride (Lin and Garcia, 2012). Briefly, 60 µg of histone extract was mixed with 100 mM NH_4_HCO_3_, pH 8.0, and concentrated to a volume of 20–30 µL. Next, the same volume of propionylation solution (propionic anhydride in 2-propanol, 1:3) was added and the pH was adjusted to 8.0 with NH_4_OH. The samples were incubated at 37 °C for 15 min, concentrated to a volume of 5–10 µL in a SpeedVac, diluted to 30 µL with ddH_2_O and the propionylation reaction was repeated with fresh propionylation solution. Propionylated histone extracts were digested according to a previously described protocol (Lin and Garcia, 2012). Briefly, 20 µg of propionylated histones were mixed with 15 µL of 100 mM NH_4_HCO_3_ and diluted with ddH_2_O to a final volume of 50–100 µL. Trypsin was added to the histone extracts at a 1:20 (enzyme:protein) ratio, and the samples were incubated at 37 °C for 16 h. The digestion reaction was stopped by adjusting the pH to 3.0 with acetic acid, and the samples were concentrated to approximately 10% of the original volume in a SpeedVac. Subsequently, the peptides were desalted onto StageTips C18 (Rappsilber et al, 2007) for analysis by nanoLC-MS/MS.

### 2.5 NanoLC-MS/MS analysis

The nanoLC-MS/MS analysis of the peptide mixtures was performed as previously described (de Almeida et al., 2021). Briefly, the peptides were eluted from the C18 StageTips with 0.1% formic acid, 5% DMSO, and 5% ACN, separated by nano-liquid chromatography (nanoLC), and analyzed by electrospray ionization tandem mass spectrometry (ESI-MS/MS) using a UHPLC Easy-nLC 1000 (Thermo Scientific, Waltham, MA, USA) coupled to an LTQ Orbitrap XL (Thermo Scientific, Waltham, MA, USA). Peptides were separated on a 30 cm × 75 µm inner diameter column packed with C18 reversed-phase resin (ReproSil-PurC18-AQ1.9 µm, Dr. Maisch GmbH, Ammerbuch-Entringen, Germany), maintained at a constant temperature of 60 °C. Solvent A consisted of 0.1% formic acid and 5% DMSO in water, whereas solvent B consisted of 0.1% formic acid and 5% DMSO in ACN. The gradient ranged from 5 to 40% solvent B over 128 min. The mass spectrometer was operated in data-dependent acquisition (DDA) mode, and the five most intense precursor ions with charge states ≥2 detected in each MS1 scan were fragmented by CID and HCD. Two technical replicates of each sample were analyzed.

### 2.6 Identification of peptides and hPTMs

Peptides and hPTM sites were identified and quantified through automated database searches using the PEAKS Studio X (v. 10) computational proteomics platform (Ma et al., 2003). The PEAKS DB (Zhang et al., 2012) and PEAKS PTM (Han et al., 2011) algorithms were used to identify peptides and hPTMs. Proteins and hPTMs were searched against a database containing 20,257 *T. cruzi* Dm28c “2017” sequences downloaded from TriTrypDB (Shanmugasundram et al., 2023) on August 15, 2018. For both PEAKS DB and PEAKS PTM searches, mass tolerances of 10 ppm for precursor ions and 0.5 Da for fragment ions were used. Arg-C was specified as the digestion enzyme using specific digestion mode. A minimum peptide length of five amino acids was used, allowing up to two missed cleavages. Carbamidomethylation was specified as a fixed PTM in PEAKS DB, whereas the variable modifications included monomethylation (K/R), dimethylation (K/R), trimethylation (K), acetylation (K), acetylation (N-term), propionylation (K), propionylation (N-term), methylpropionylation (K), phosphorylation (STY), and oxidation (M). For PEAKS PTM, the following additional modification types were included: monomethylation (N-term), dimethylation (N-term), glutarylation (K), 2-hydroxyisobutyrylation (K), hydroxylation (Y), malonylation (K), succinylation (K), ubiquitination (K), crotonylation (K), citrullination (R), methylation (Q), phosphorylation (H), butylation (K), acetylation (S/T), formylation (K), and deamidation (N/Q). Peptide and protein identifications were filtered at a 1% false discovery rate (FDR). An Ascore ≥20 was used to validate the localization of hPTM sites. For subsequent analyses, the histone sequence presenting the highest identification score and the greatest number of identified hPTMs within each histone family was selected as the reference sequence. These selected histones have the following identifiers in the TriTrypDB database: H2A (BCY84_17443), H2B (BCY84_06291), H3 (BCY84_02640), H4 (BCY84_15632), H2A.Z (BCY84_22061), H2B.V (BCY84_04421), H3.V (BCY84_18558), and H1 (BCY84_22685).

### 2.7 Quantitative analysis of hPTM marks

Histone-modified peptides identified under E, S, NS-2h, and NS-6h in triplicate, for each analytical strategy (LC-MS/MS and GeLC-MS/MS) were analyzed for differential modulation between conditions. The intensity of each hPTM-containing peptide was normalized to the intensity of the corresponding histone protein. For each histone, a normalization factor (protein ratio) was calculated as the LFQ intensity of that histone in each sample divided by the average LFQ intensity of the same histone across all samples (**Supplementary Tables 1**, **2**). Each hPTM-containing peptide was then normalized to the abundance of its corresponding histone by dividing its LFQ intensity by the protein ratio calculated for that histone in the same sample. Then, a single normalized LFQ value for each hPTM-containing peptide was obtained by averaging the technical injection duplicates from the mass spectrometer for each biological replicate (**Supplementary Tables 3**, **4**). For the global analysis, normalized intensities of peptides carrying the same modification type were summed and log2-transformed prior to comparison across conditions using ANOVA (FDR 5%) in Perseus v.1.6.15.0 (Tyanova et al., 2016). For the analysis of individual hPTM-containing peptides, normalized LFQ intensities of each peptide were log2-transformed, and peptides detected in at least two biological replicates per condition were retained for differential modulation analysis. ANOVA with FDR 5% (q-value) was applied, and p-values were adjusted using a permutation-based method with 250 randomizations to control the FDR (Tyanova and Cox, 2018). Pairwise comparisons between biological conditions were subsequently performed using Tukey’s Honest Significant Difference test (FDR 5%). Differentially modulated peptides were hierarchically clustered using Euclidean distance. In addition, relative site occupancy was estimated by dividing the intensity of the modified peptide by the sum of the intensities of the corresponding modified and unmodified forms of the peptide (**Supplementary Tables 5**, **6**).

## 3 Results

### 3.1 Identification of canonical, variant, and linker histones and their hPTMs

*T. cruzi* epimastigotes representing the exponential growth phase (E), stationary growth phase (S), nutritional stress for 2 h (NS-2h), and nutritional stress for 6 h (NS-6h) were processed according to the experimental workflow shown in **Figure 1**. Qualitative analysis by SDS-PAGE showed that histone enrichment by acid extraction was efficient under all conditions, with a clear predominance of histone bands in the region between 12 and 15 kDa, resulting in a less complex, histone-enriched extract compared to the whole-cell extract (**Figure 2**). Similar enrichment profiles were observed across all four conditions, indicating consistent histone extraction throughout the experimental workflow.

**Figure 2:**
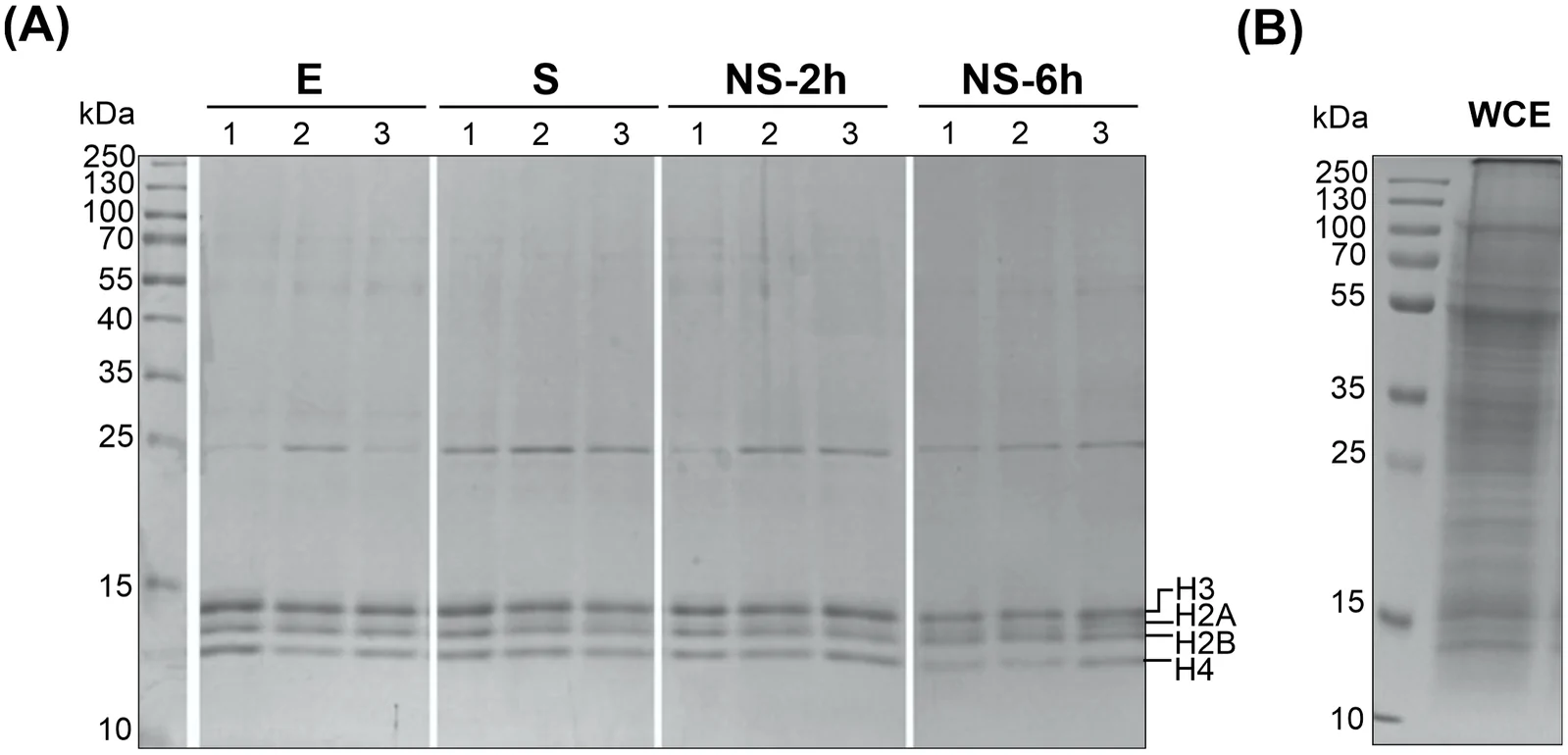
Qualitative analysis of histone enrichment from *T. cruzi* epimastigotes under exponential growth, stationary growth, and nutritional stress. **(A)** SDS-PAGE analysis of histone-enriched extracts from parasites in the exponential growth (E), stationary growth (S), and nutritional stress for 2 h (NS-2h) and nutritional stress for 6 h (NS-6h). Histones are indicated between approximately 12 and 15 kDa. Equal amounts of protein (10 µg/lane) were resolved by 15% SDS-PAGE and stained with Coomassie Brilliant Blue R250. The molecular weight (kDa) marker was PageRuler™. **(B)** Whole-cell extract (WCE; 10 µg) from *T. cruzi* epimastigotes was analyzed under the same conditions for comparison.

Histone-enriched extracts were subsequently analyzed using two complementary proteomic strategies - GeLC-MS/MS and LC-MS/MS - to characterize the hPTM landscape of *T. cruzi* under the different growth and nutritional stress conditions. Overall, both analytical strategies generated robust identification metrics, including the numbers of peptide-spectrum matches (PSMs), peptides, proteins, and protein groups, across all biological conditions (**Figure 3A**). Canonical, variant, and linker histones were successfully identified using both analytical strategies and showed similar sequence coverage across the biological conditions (**Figures 3B, C**). The number of non-redundant histone peptides and their corresponding modified peptide versions identified by each analytical strategy are summarized in **Supplementary Tables 7**, **8**. Combining both analytical strategies resulted in the identification of 172 non-redundant hPTMs across the four growth and nutritional stress conditions (**Figure 3D**; **Supplementary Table 9**). Each strategy identified unique hPTMs, whereas 79 were shared between LC-MS/MS and GeLC-MS/MS, demonstrating the complementarity of the two analytical approaches (**Figure 3E**). Thus, hPTMs detected by both workflows reinforced confidence in their identification, whereas hPTMs detected by only one workflow expanded the overall coverage of the hPTM landscape. In addition, a total of 67 hPTMs were identified in all four biological conditions evaluated, whereas additional hPTMs were either unique to or shared among specific conditions (**Figure 3F**).

**Figure 3:**
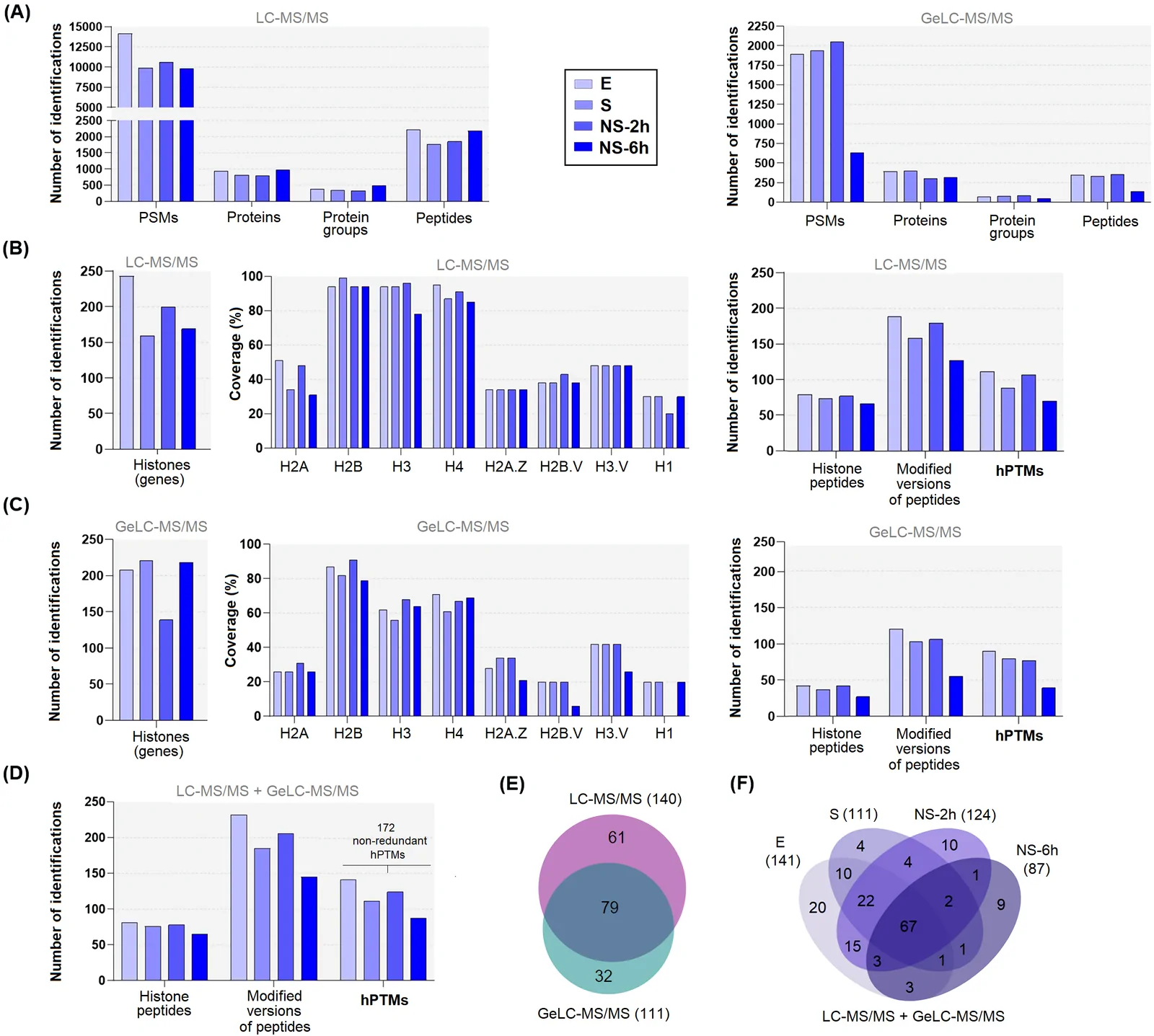
Identification of histones and histone post-translational modifications (hPTMs) in *T. cruzi* epimastigotes under exponential growth, stationary growth, and nutritional stress. **(A)** Numbers of peptide-spectrum matches (PSMs), proteins, protein groups, and peptides identified by LC-MS/MS and GeLC-MS/MS. **(B, C)** Numbers of identified canonical, variant, and linker histones, sequence coverage of representative histones selected for downstream analyses, and the corresponding numbers of histone peptides, modified peptide versions, and hPTMs identified by LC-MS/MS and GeLC-MS/MS, respectively. **(D)** Numbers of non-redundant histone peptides, modified peptide versions, and hPTMs identified under each biological condition after combining both analytical strategies. **(E)** Overlap of non-redundant hPTMs identified by LC-MS/MS and GeLC-MS/MS. **(F)** Distribution of non-redundant hPTMs across the four experimental conditions. Bar colors indicate parasites in the exponential growth **(E)**, stationary growth (S), nutritional stress for 2 h (NS-2h) and nutritional stress for 6 h (NS-6h).

### 3.2 Global and site-specific modulation of histone post-translational modifications

The identified hPTMs were subsequently evaluated according to their normalized intensity (**Figure 4**) and relative site occupancy (**Figure 5**), providing complementary measures of hPTM modulation across the biological conditions and proteomic strategies. Distinct modulation profiles were observed among individual hPTMs. Several hPTMs were identified in all four conditions analyzed by both strategies, including H2A.ZK54ac/K58ac, H2BA1me, H3K76me3, and H3.VK94me3. The T24–K34 region of H2B displayed consistently high normalized intensities. Although most hPTM sites showed low relative site occupancy, some of these sites detected in all conditions also exhibited high relative site occupancy for specific modifications across all four biological conditions and in both strategies, including H2A.ZK58ac, H2BA1me, H2BK34me, H3K76me3, and H3.VK94me3. Additional notable examples included the modifications at the H2BK48 site (ac, cro, glu, and hib) and the hyper-acetylated N-terminal region of H2A.Z (identified under all conditions except E in the GeLC-MS/MS strategy). Twenty-three novel hPTM sites were identified compared with those previously described and compiled from *T. cruzi* epimastigotes in the exponential growth phase (de Almeida et al., 2021) (underlined in **Figures 4**, **5**). Of these, 15 were identified under conditions other than the exponential phase.

**Figure 4:**
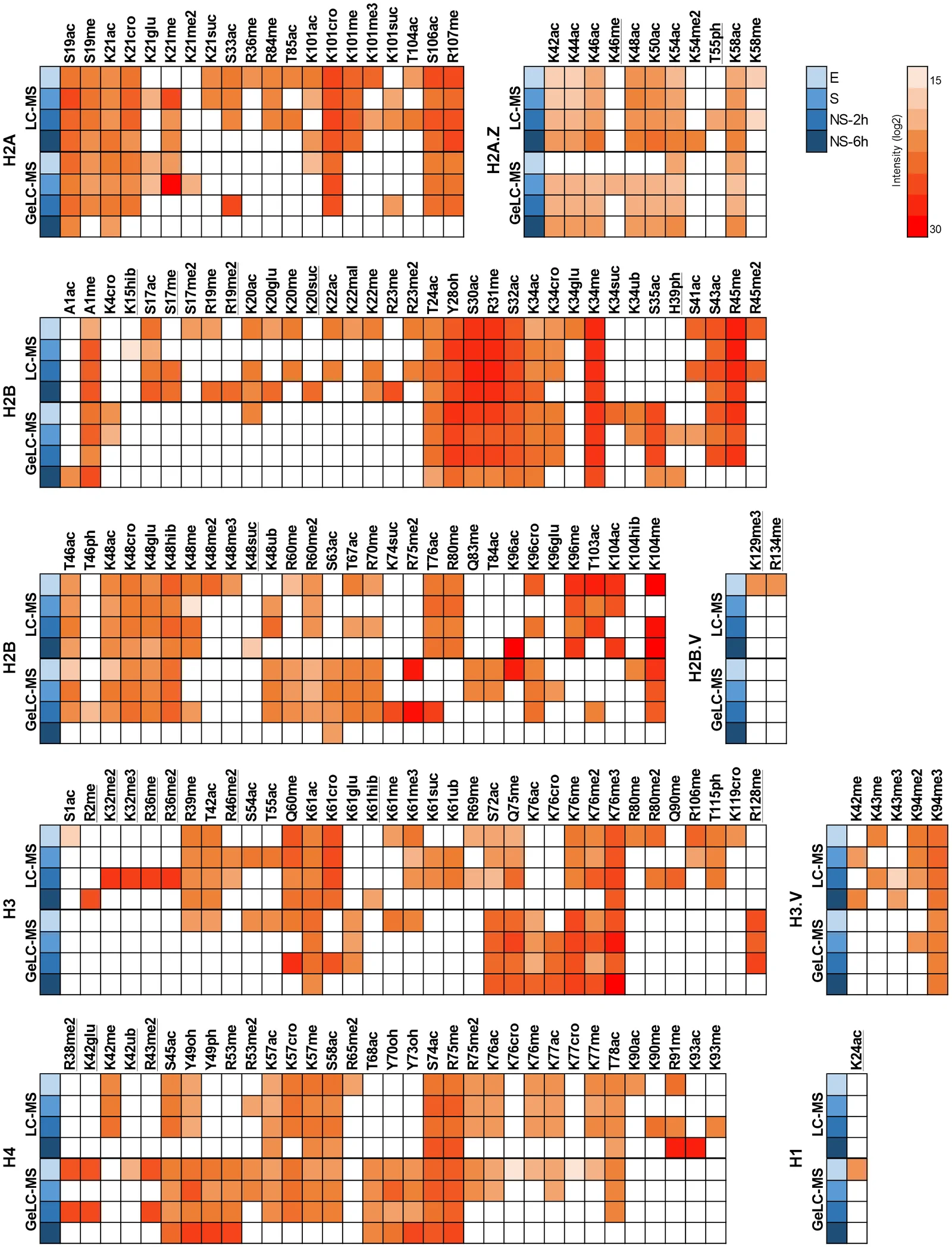
Normalized intensity of histone post-translational modifications (hPTMs) identified in *T. cruzi* epimastigotes under different growth and nutritional stress conditions. For each reference histone, LC-MS/MS and GeLC-MS/MS results are displayed and columns represent the four experimental conditions: exponential growth (E), stationary growth (S), and nutritional stress for 2 h (NS-2h) and nutritional stress for 6 h (NS-6h). Rows correspond to individual hPTM sites. The intensity of each modified hPTM-containing peptide was normalized to the abundance of its corresponding reference histone, as described in the Materials and Methods. Underlined hPTM sites (n = 23) indicate novel modification sites not previously reported in *T. cruzi* histones (de Almeida et al., 2021). The PTM abbreviations are acetylation (ac), monomethylation (me), dimethylation (me2), trimethylation (me3), phosphorylation (ph), crotonylation (cro), glutarylation (glu), succinylation (suc), 2-hydroxyisobutyrylation (hib), malonylation (mal), hydroxylation (oh), and ubiquitination (ub).

**Figure 5:**
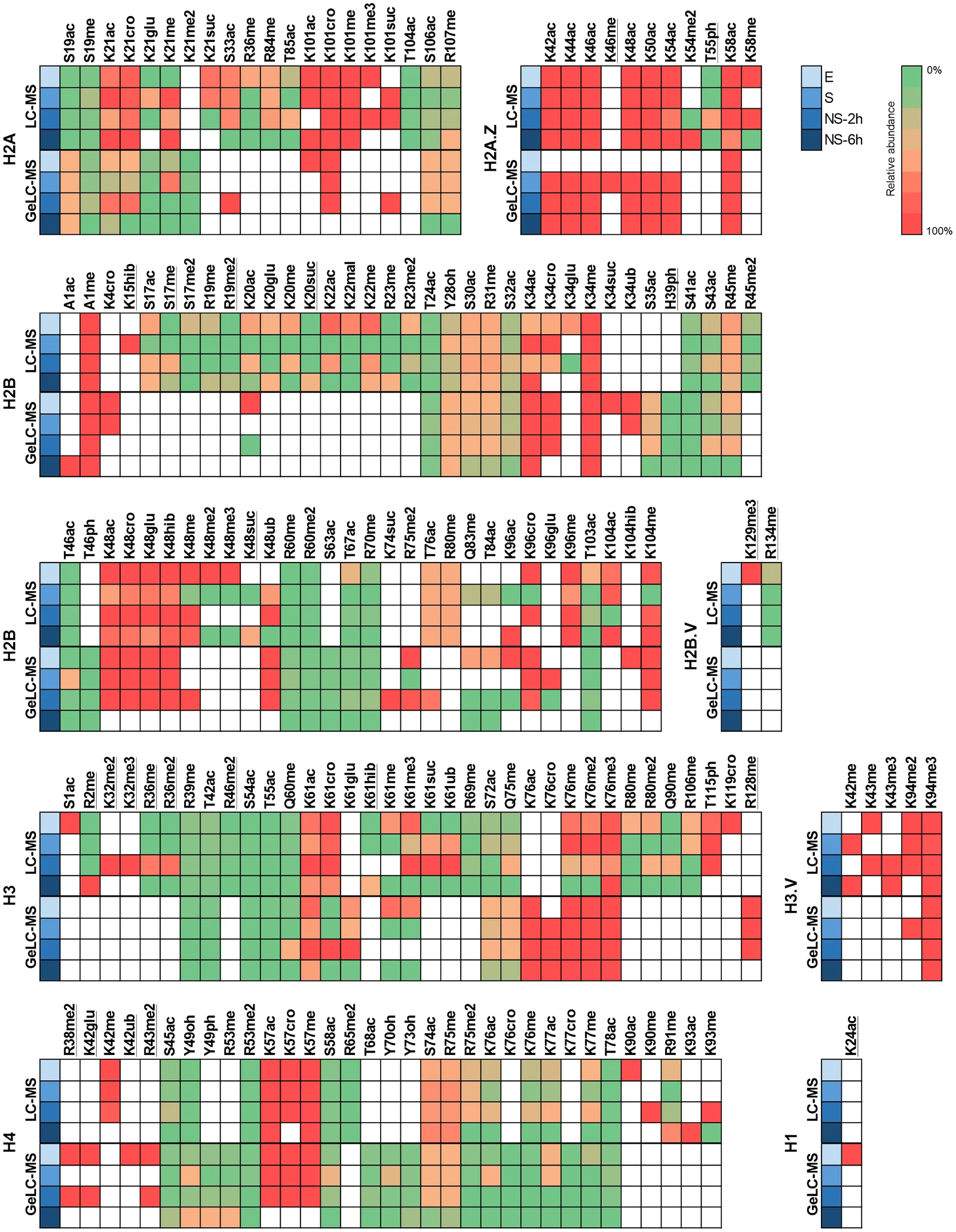
Relative site occupancy of histone post-translational modifications (hPTMs) identified in *T. cruzi* epimastigotes under different growth and nutritional stress conditions. For each reference histone, LC-MS/MS and GeLC-MS/MS results are displayed and columns correspond to the four experimental conditions: exponential growth (E), stationary growth (S), nutritional stress for 2 h (NS-2h) and nutritional stress for 6 h (NS-6h). Relative site occupancy was estimated by dividing the intensity of each hPTM-containing peptide by the sum of the intensities of the corresponding modified and unmodified forms of the peptide. Color intensity reflects the estimated relative site occupancy. Values of 100% indicate that only the modified peptide was detected, whereas values of 0% indicate detection of only the unmodified peptide. Underlined hPTM sites (n = 23) indicate novel modification sites not previously reported in *T. cruzi* histones (de Almeida et al., 2021). The PTM abbreviations are acetylation (ac), monomethylation (me), dimethylation (me2), trimethylation (me3), phosphorylation (ph), crotonylation (cro), glutarylation (glu), succinylation (suc), 2-hydroxyisobutyrylation (hib), malonylation (mal), hydroxylation (oh), and ubiquitination (ub).

The identified hPTMs showed condition-dependent modulation in different ways, affecting both global hPTM-class abundance and individual modified peptides. Because the nutritional-stress conditions correspond to general nutrient limitation in TAU medium, rather than selective deprivation of individual nutrient classes, the modulation patterns identified here should be interpreted as stress-associated changes rather than nutrient-class-specific signatures. Global modulation of hPTM classes during parasite adaptation to nutritional stress was evaluated by comparing the normalized intensities of modified peptides across the four biological conditions. The unmodified versions of histone peptides were the most abundant, whereas modified peptides were distributed into three abundance groups according to hPTM class (**Figure 6**). Monomethylation and acetylation represented the most abundant hPTM classes and exhibited modest fluctuations across the analyzed conditions, with no significant differences detected. An intermediate-abundance group comprised crotonylation, hydroxylation, dimethylation, and trimethylation, which exhibited distinct modulation profiles across conditions. Within this group, dimethylation, trimethylation, and crotonylation exhibited significant modulation, whereas hydroxylation showed only modest variation. Trimethylation displayed a progressive increase toward prolonged nutritional stress. In contrast, crotonylation levels significantly decreased between NS-2h and NS-6h, whereas dimethylation showed significant fluctuations but no consistent temporal trend across conditions. The third abundance group included the remaining non-acetyl acylations (glutarylation, succinylation, and 2-hydroxyisobutyrylation), ubiquitination, and phosphorylation. No statistically significant changes were detected for these hPTM classes across conditions.

**Figure 6:**
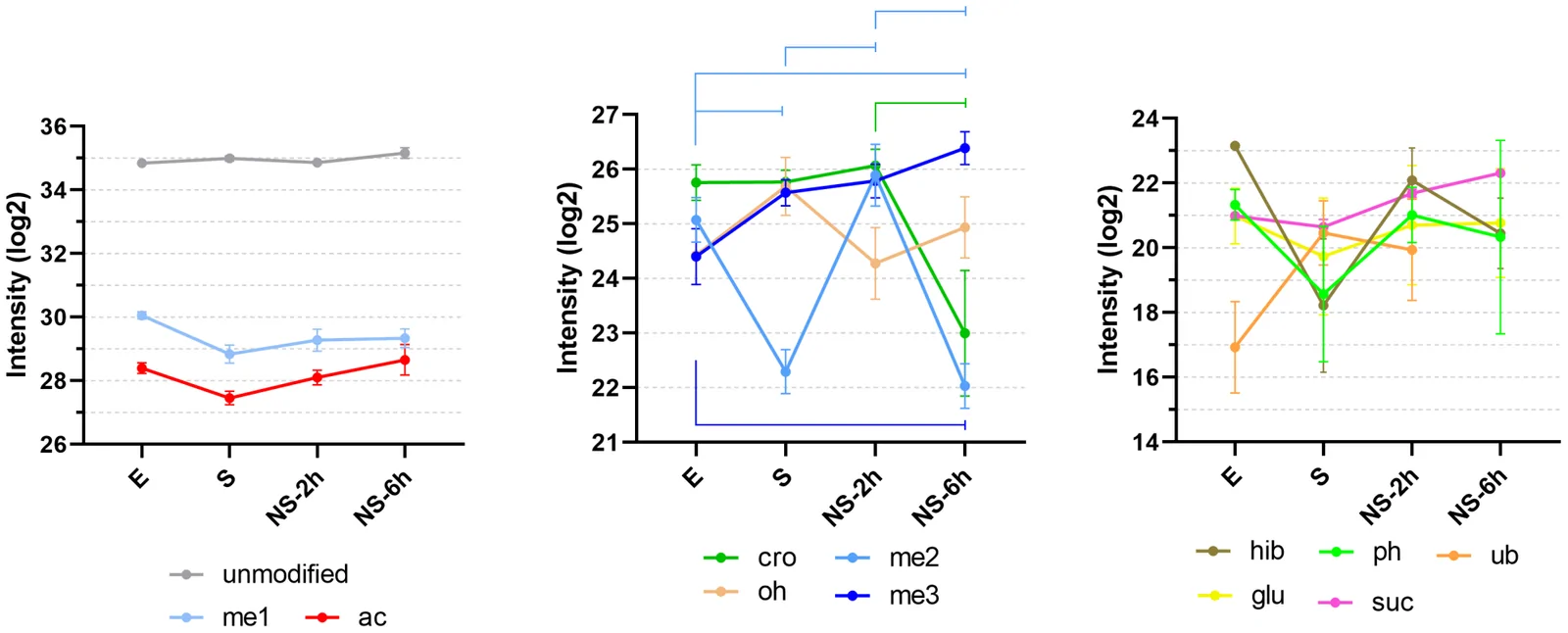
Global abundance profiles of hPTM classes identified in *T. cruzi* across exponential growth, stationary growth, and nutritional stress. Points represent the mean ± standard error of the mean (SEM) of the three biological replicates. Horizontal brackets indicate statistically significant differences between conditions (ANOVA with Tukey’s multiple-comparison test, 5% significance level). The x-axis shows parasites in exponential growth (E), stationary growth (S), nutritional stress for 2 h (NS-2h), and nutritional stress for 6 h (NS-6h). PTM abbreviations are acetylation (ac), monomethylation (me1), dimethylation (me2), trimethylation (me3), phosphorylation (ph), crotonylation (cro), glutarylation (glu), succinylation (suc), 2-hydroxyisobutyrylation (hib), hydroxylation (oh), and ubiquitination (ub).

The peptides detected in the biological replicates showed moderate to high reproducibility across all four biological conditions (**Figure 7A**). A total of 16 modified histone peptides, corresponding to 20 hPTMs, were differentially modulated across conditions. Hierarchical clustering of these peptides revealed four clusters of co-regulated modified peptides (**Figure 7B**). These peptides displayed four major temporal modulation profiles across the biological conditions (**Figure 8**). Most exhibited progressive increases in normalized intensity during nutritional stress, particularly the acetylated peptides derived from the N-terminal region of H2A.Z (amino acids 41–62), H2BT24ac, H2BK96meK104ac, and H3K61ac, all of which reached their highest levels in the NS-6h condition. In contrast, H2AK21cro, H2BK48cro, and H4T78ac showed progressive decreases toward prolonged nutritional stress. Other regulated peptides, including H2BY28oh, H2BS30acR31me, H2AS19ac, and H3.VK94me3, displayed transient modulation patterns throughout the experiment, whereas H3K76me2 exhibited a biphasic profile, with a marked decrease after 2 h of nutritional stress followed by a pronounced increase after 6 h of nutritional stress (NS-6h).

**Figure 7:**
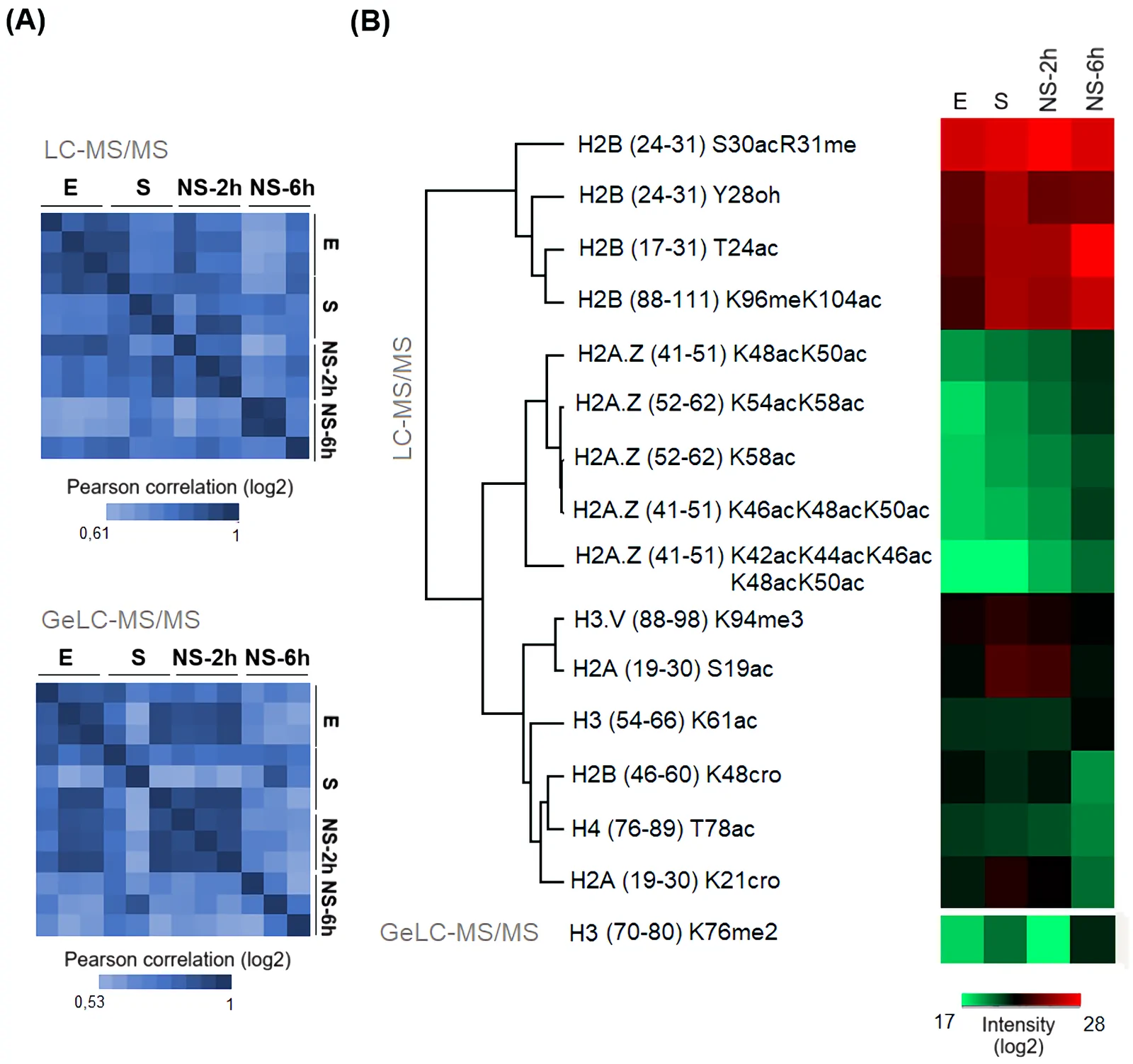
Quantification and clustering of *T. cruzi* histone-modified peptides across exponential growth, stationary growth, and nutritional stress. **(A)** Pearson correlation matrices based on normalized peptide intensities obtained from three biological replicates of parasites in exponential growth (E), stationary growth (S), nutritional stress for 2 h (NS-2h), and nutritional stress for 6 h (NS-6h) using LC-MS/MS and GeLC-MS/MS analytical strategies. **(B)** Hierarchical clustering of differentially modulated histone-modified peptides. PTM abbreviations are acetylation (ac), monomethylation (me), dimethylation (me2), trimethylation (me3), crotonylation (cro), and hydroxylation (oh).

**Figure 8:**
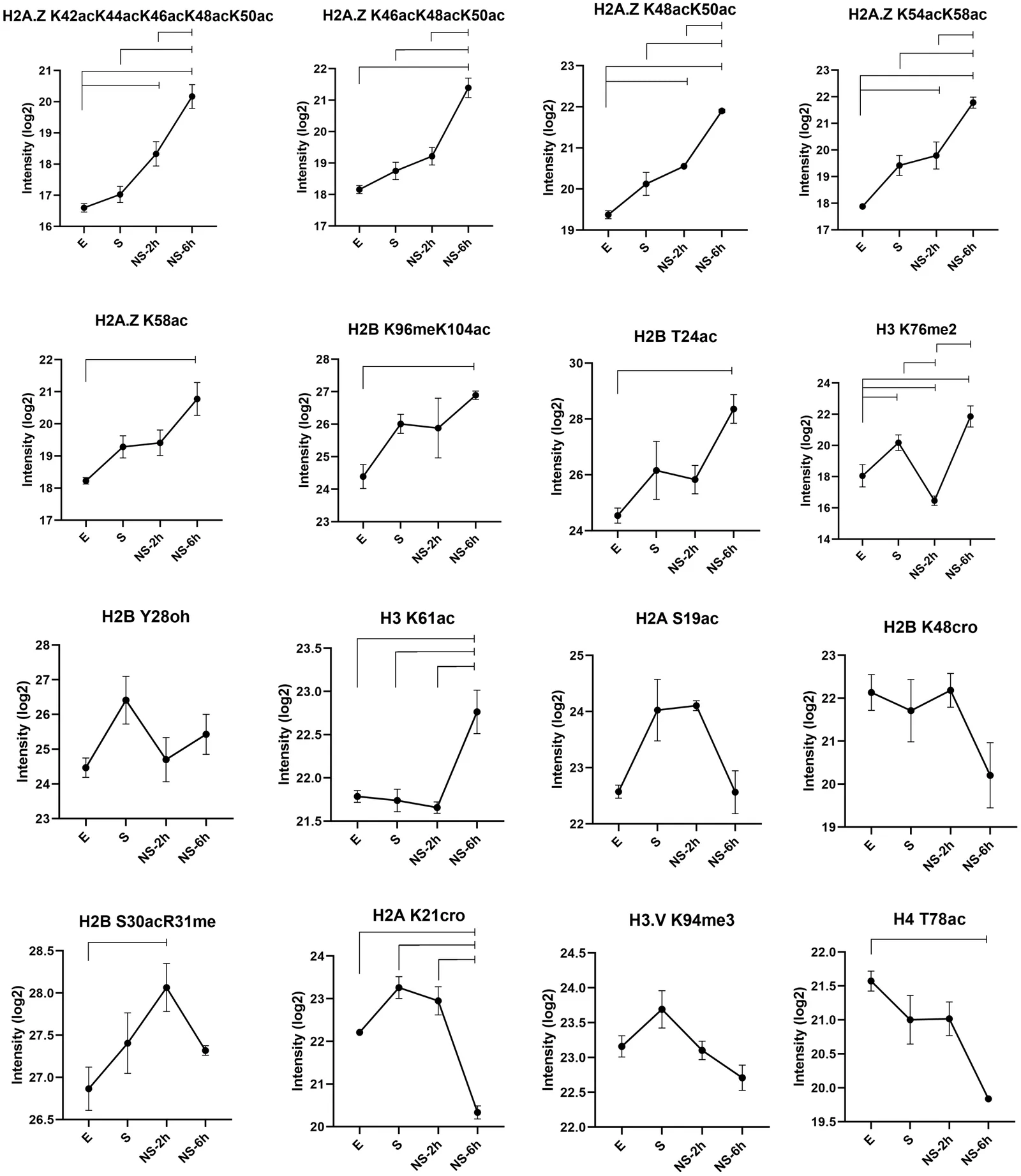
Differentially regulated hPTMs across exponential growth, stationary growth, and nutritional stress. Error bars represent the standard error of the mean (SEM) of the three biological replicates. Horizontal brackets indicate statistically significant differences between conditions (ANOVA with Tukey’s multiple-comparison test, 5% significance level). The x-axis shows parasites in exponential growth (E), stationary growth (S), nutritional stress for 2 h (NS-2h), and nutritional stress for 6 h (NS-6h). The PTM code is formatted as “amino acid-position-modification” and the PTM abbreviations are acetylation (ac), monomethylation (me), dimethylation (me2), trimethylation (me3), crotonylation (cro), and hydroxylation (oh).

## 4 Discussion

Nutritional stress is one of the key physiological signals associated with the onset of *T. cruzi* metacyclogenesis (Figueiredo et al., 2000), during which epimastigotes undergo extensive changes in gene expression and differentiate into infective metacyclic trypomastigotes. Although hPTMs have previously been mapped in *T. cruzi* epimastigotes (Picchi et al., 2017; de Almeida et al., 2021), how these modifications are modulated in epimastigotes under nutrient-limited conditions and which of them may participate in this early adaptive transition has remained largely unknown. By quantitatively characterizing the hPTM landscape as parasites progress from optimal growth conditions to prolonged nutritional stress, the present study provides insights into the dynamic global and site-specific hPTM remodeling associated with nutrient limitation and provides a resource for future functional studies aimed at understanding the role of hPTMs in epimastigote adaptation to nutritional stress.

The combination of LC-MS/MS and GeLC-MS/MS generated an extensive and consistent hPTM dataset that supported the evaluation of hPTM dynamics across the different biological conditions. Together, the two analytical strategies expanded the currently described repertoire of *T. cruzi* histone modifications by identifying previously unreported hPTM sites. Importantly, the analytical strategies proved highly complementary, with each contributing unique hPTMs, demonstrating that their combined use maximized hPTM identification and sequence coverage, similar to what was previously described (Picchi et al., 2017; de Almeida et al., 2021). Overall, these data provided a robust basis for detecting both constitutive and dynamically regulated hPTMs across the transition from optimal growth to nutritional stress.

Mapping hPTMs across the four biological conditions revealed a heterogeneous landscape in terms of detection, intensity, and relative site occupancy, composed of both widely detected and condition-dependent modifications. Several hPTMs were consistently detected by both analytical strategies throughout the transition from exponential growth to prolonged nutritional stress, suggesting that they represent stable features of the *T. cruzi* hPTM landscape and may play important roles in parasite biology, which remain to be investigated through targeted functional studies. Although their widespread detection may, in part, reflect their higher intensity and/or occupancy, a subset of low-intensity and low-occupancy modifications were also consistently detected, indicating that these sites may also represent relevant candidates for targeted functional studies. Representative examples include H2A.ZK54ac and H2A.ZK58ac, previously reported in different studies of *T. cruzi* (Picchi et al., 2017; de Almeida et al., 2021; de Jesus et al., 2016; de Lima et al., 2020), H2BA1me, previously identified in *T. brucei* (Janzen et al., 2006), H3K76me3, which is present throughout the *T. cruzi* cell cycle and functions as a checkpoint of cell-cycle progression (Nunes et al., 2020), and H3.VK94me3, which corresponds to H3K76 based on sequence alignment and may represent its functional counterpart. The combined evaluation of normalized intensity and relative site occupancy also highlighted distinct patterns among individual hPTMs, including the consistently high normalized intensities in the H2B T24–K34 region, the multiple modifications identified at H2BK48 (ac, cro, glu, and hib), and the hyper-acetylated N-terminal region of H2A.Z, which has been associated with transcription start regions and bromodomain factors in trypanosomatids (Siegel et al., 2009; Yang et al., 2017). Together, these observations identify candidate hPTMs and histone regions for future functional studies aimed at understanding epigenetic regulation during parasite adaptation.

Since the experimental design did not include selective deprivation of individual nutrient classes, the observed hPTM changes cannot be attributed to depletion of specific nutrients such as amino acids or carbohydrates. Instead, they should be interpreted as candidate hPTM responses associated with the general TAU-induced nutrient-limitation model used here. Despite the distinct abundance levels observed among the 12 hPTM classes identified, the global abundance of most hPTM classes remained remarkably stable throughout the transition from exponential growth to prolonged nutritional stress. This observation suggests that the early stages of parasite adaptation are associated primarily with selective remodeling affecting a subset of hPTM classes and individual modification sites, rather than broad remodeling of the overall hPTM landscape. Interestingly, the only hPTM classes exhibiting significant global modulation belonged to the intermediate-abundance group. Dimethylation showed significant fluctuations without a consistent temporal trend, trimethylation progressively increased during nutritional stress, whereas crotonylation significantly decreased after prolonged nutritional stress. Unlike the findings reported by de Lima and colleagues, in which monomethylation showed lower overall levels than the other methylation states and acetylation (de Lima et al., 2020), monomethylation represented the predominant methylation state in our dataset. These differences may reflect the distinct biological conditions evaluated in the two studies. The remaining non-acetyl acylations and phosphorylation were consistently detected at lower abundance. The low abundance of non-acetyl acylations agrees with observations in other eukaryotes and may reflect the limited availability of their corresponding acyl-CoA substrates (Sabari et al., 2017). Phosphorylation, on the other hand, is a transient and unstable modification (Zhao and Jensen, 2009) that generally requires dedicated enrichment strategies for efficient detection. Because no phosphopeptide enrichment was performed in the present work, its low abundance was expected. Likewise, the overall lower abundance of modified peptides relative to their unmodified counterparts is consistent with the generally low stoichiometry of hPTMs. Consequently, the abundance of each hPTM class should be interpreted in the context of the analytical dynamic range of detection of the peptides carrying those modifications, since no PTM-specific enrichment was performed.

Site-specific analysis revealed that nutritional stress was associated with coordinated remodeling of a limited set of modified histone peptides. Among the regulated peptides, the most prominent pattern involved the N-terminal region of H2A.Z, particularly peptides spanning amino acids 42–58. These peptides contained different combinations of acetylated lysines, but consistently increased in intensity during nutritional stress, with the strongest differences observed after prolonged nutritional stress. This result is particularly relevant because H2A.Z is closely associated with gene regulation in eukaryotes, especially in its acetylated form (Giaimo et al., 2019). Studies in *T. brucei* provide a useful framework for interpreting this finding, H2A.Z dimerizes with H2B.V (Lowell et al., 2005), and the H2A.Z hyperacetylated N-terminal region binds bromodomain factor 2 (TbBDF2) (Yang et al., 2017). Also, the enrichment of H2A.Z and H2B.V has been observed at transcription initiation regions (Siegel et al., 2009), where it likely contributes to reduced nucleosome stability and increased accessibility to the transcriptional machinery. In *T. cruzi*, although interactome assays show that H2B.V associates with H2A.Z, bromodomain factor 2 and is enriched near putative transcriptional start regions (Rosón et al., 2022), whether acetylation of the H2A.Z N-terminal region contributes to these interactions or related chromatin functions has not been specifically investigated. Thus, the modulation of H2A.Z acetylation observed here identifies a candidate chromatin-associated event in *T. cruzi*, while its possible relationship with chromatin accessibility, transcriptional regulation, or bromodomain-mediated recognition remains to be directly investigated. Other regulated peptides showed distinct temporal profiles, including transient modulation of H2B S30acR31me and progressive decreases in H2AK21cro, H2BK48cro, and H4T78ac. Together, these findings indicate that nutritional stress is associated with selective remodeling of specific hPTMs, rather than uniform changes across the hPTM landscape. Future studies using independent validation strategies, including antibody-based assays with suitable specificity for acetylated *T. cruzi* H2A.Z or for other relevant modified residues identified here, will be important to confirm this modulation and explore its functional relevance.

Taken together, these findings indicate that nutritional stress is associated with selective remodeling of histone post-translational modifications, pointing to hPTM remodeling as a candidate chromatin-associated feature of the early adaptation of *T. cruzi* to nutrient limitation. By moving beyond the cataloging of hPTMs to characterize their global and site-specific dynamics during this transition, this study establishes a framework for hypothesis-driven functional studies. Although the hPTM changes identified here cannot yet be directly linked to metacyclic trypomastigote formation or infectivity, this dataset provides a resource for future studies aimed at determining whether nutritional-stress-associated hPTM remodeling contributes to metacyclogenesis-associated differentiation. Regarding possible drug-development implications, this study does not directly identify therapeutic targets; however, the regulated hPTMs described here may help prioritize chromatin-associated factors for functional validation. In particular, the increased abundance of acetylated peptides from the N-terminal region of H2A.Z during nutritional stress highlights this histone region as a candidate for studies aimed at identifying associated reader, writer, and eraser proteins. Determining whether these factors contribute to parasite survival, differentiation, or infectivity will be necessary before their therapeutic relevance can be assessed.

## Data Availability

The mass spectrometry proteomics data have been deposited to the ProteomeXchange Consortium via the PRIDE (Perez-Riverol et al., 2025) partner repository with the dataset identifier PXD075740.
